# Antimicrobial resistance and genomic characterization of *Salmonella* Minnesota, ESBL-producing *Escherichia coli*, and ESKAPE pathogens isolated from Brazilian broiler chicken carcasses

**DOI:** 10.3389/fmicb.2026.1917698

**Published:** 2026-09-15

**Authors:** Isabela Pádua Zanon, Rafaela Assis Machado, Thayanne Gabryelle Viana de Souza, Bruno Leandro de Almeida Brito, Tales Fernando da Silva, Tadeu Chaves de Figueiredo, Marcelo Resende de Souza, Júlio César Câmara Rosa, Henrique César Pereira Figueiredo, Rodrigo Otávio Silveira Silva, Silvana de Vasconcelos Cançado

**Affiliations:** 1 Escola de Veterinária, Universidade Federal de Minas Gerais (UFMG), Belo Horizonte, Brazil; 2 Ministério da Agricultura e Pecuária, Brasília, Brazil

**Keywords:** chicken meat, multidrug-resistance, *salmonellosis*, AMR, *AmpC*

## Abstract

**Introduction:**

Brazil is the world’s largest exporter of chicken meat, and poultry products are recognized as important reservoirs of foodborne pathogens. *Salmonella enterica* serovar Minnesota has emerged as the predominant serovar in the poultry industry and is frequently associated with multidrug-resistant strains carrying multiple antimicrobial resistance genes. In addition, *Escherichia coli* and ESKAPE pathogens have been increasingly isolated from poultry products worldwide, representing a growing public health concern due to their antimicrobial resistance and dissemination potential. This study aimed to characterize the antimicrobial resistance profiles of *Salmonella* spp., *E. coli*, and ESKAPE pathogens isolated from chicken carcasses using phenotypic and genomic approaches.

**Methods:**

The study was conducted in a poultry slaughterhouse inspected by the Brazilian Ministry of Agriculture that supplies both the domestic and international markets. Thirty carcasses with visible gastrointestinal contamination and 30 without visible contamination were sampled. To investigate the epidemiology of ESBL-producing strains, representative isolates with distinct resistance profiles were subjected to whole-genome sequencing (*n* = 9).

**Results:**

A total of 48 *Salmonella* spp. and 99 *E. coli* isolates were recovered. Among the ESKAPE pathogens, *Klebsiella pneumoniae* (*n* = 68), *Enterobacter* spp. (*n* = 53), *Pseudomonas aeruginosa* (*n* = 59), *Acinetobacter baumannii* (*n* = 14), and Staphylococcus aureus (*n* = 10) were also isolated. *Salmonella* Minnesota was the predominant serovar. Members of the Enterobacterales exhibited high frequencies of multidrug resistance and extended-spectrum β-lactamase (ESBL) production, detected in 52.1% of *Salmonella* spp., 56.6% of *E. coli*, and 13.2% of *K. pneumoniae* isolates. Genomic analysis identified clinically relevant resistance determinants, including *bla*_CMY-2_ in *Salmonella* spp. and ESBL-associated genes such as *bla*_CTX-M-15_ and *bla*_CTX-M-55_ in *E. coli* and *K. pneumoniae*. MLST identified internationally disseminated high-risk clones, including *E. coli* ST38 and *K. pneumoniae* ST307, while SNP analysis demonstrated close genetic relatedness to isolates recovered from human infections.

**Discussion:**

These findings highlight the potential role of chicken products as reservoirs of clinically relevant multidrug-resistant bacteria and reinforce the importance of continuous antimicrobial resistance surveillance throughout the poultry production chain.

## 1 Introduction

Global chicken meat production reached 128 million tons in 2024, surpassing pork to become the most widely produced meat worldwide (FAO, 2024). In addition, poultry is currently the most consumed animal meat globally (Connolly et al., 2022), and it is projected to account for 45% of the protein derived from all meat sources by 2034 (OECD and Food and Agriculture Organization of the United Nations, 2025). In this context, Brazil ranks among the leading chicken meat producers worldwide and is the largest exporter (FAS, 2026). Given its central importance in global food supply and trade, this scenario raises concerns regarding food safety, particularly the dissemination of foodborne pathogens and associated antimicrobial resistance.

Poultry has been widely associated with foodborne pathogens, representing a major public health concern (on behalf of the RIBMINS Cost Action et al., 2021). Foodborne diseases cause approximately 600 million cases and 420,000 deaths each year worldwide (Organisation mondiale de la santé, 2015). Moreover, beyond the risks related to pathogen transmission, the increasing emergence of antimicrobial resistance further exacerbates this issue due to its significant impact on both human and animal health (Rabello et al., 2020; Mulchandani et al., 2023). In fact, the emergence of multidrug-resistant strains has been widely reported worldwide and is recognized by the WHO as a major threat to human health (Organization, W. H, 2025), highlighting the critical role of surveillance programs in mitigating the spread of resistant bacteria along the food chain.

Among the resistant bacteria of public health concern, ESKAPE pathogens (*Enterococcus faecium*, *Staphylococcus aureus*, *Klebsiella pneumoniae*, *Acinetobacter baumannii*, *Pseudomonas aeruginosa*, and *Enterobacter* spp.) have emerged as a major issue in antimicrobial resistance surveillance due to their high clinical relevance and multidrug-resistant profiles (De Oliveira et al., 2020; Miller and Arias, 2024). ESKAPE bacteria have the ability to acquire and disseminate antimicrobial resistance determinants, leading to an increase in human infections and treatment failures (Kelly et al., 2024; Zeng et al., 2026). In this context, the WHO established a global priority pathogens list comprising bacteria that critically require the development of novel antimicrobials, including the ESKAPE group (WHO, 2024). In the poultry production chain, ESKAPE pathogens have been increasingly reported, raising concerns about their dissemination along the food chain and potential transmission to humans through contaminated poultry products (Leverstein-van Hall et al., 2011; Savin et al., 2020; Mak et al., 2022; Arbab et al., 2026). Therefore, monitoring these pathogens in poultry systems is essential to mitigate the spread of antimicrobial resistance.

Thus, this study aimed to characterize the antimicrobial resistance profiles of *Salmonella* spp., *E. coli*, and ESKAPE pathogens isolated from chicken carcasses produced for commercialization in both the Brazilian and international markets. In addition, selected isolates with clinically relevant antimicrobial resistance profiles were subjected to whole-genome sequencing to further characterize their sequence types and antimicrobial resistance determinants.

## 2 Materials and methods

### 2.1 Sample collection

The study comprised *Salmonella* strains, *Escherichia coli* and ESKAPE group pathogens isolated from broiler carcasses obtained at a slaughterhouse in the metropolitan region of Belo Horizonte, Minas Gerais, Brazil. All carcasses were collected immediately after the automated evisceration system. The facility is inspected by the Federal Inspection Service of the Brazilian Ministry of Agriculture. The chicken meat produced is intended for commercialization either in the Brazilian and in the foreign consumer market.

The sampling design was based on the study previously reported by Brito et al. (2025). A total of 60 broiler carcasses were collected immediately after evisceration over six consecutive weeks, with 10 carcasses sampled each week. In each sampling week, five carcasses showing visible gastrointestinal contamination and five carcasses without visible contamination were intentionally selected based on visual inspection performed. Therefore, contaminated carcasses were not randomly selected. Sampling was carried out at different times during each processing shift (beginning, middle, and end) to represent routine processing conditions. Each carcass was considered an independent experimental unit. Carcasses showing bile contamination were excluded from the study. Gastrointestinal contamination was defined as the presence of visible contents originating from the esophagus, crop, proventriculus, gizzard, or intestines on the carcass surface. From each carcass, up to three colonies displaying the same colony morphology were selected for identification. A total of 48 *Salmonella* sp. and 99 *Escherichia coli* isolates were recovered. The identified ESKAPE pathogens comprised *Klebsiella pneumoniae* (*n* = 68), *Enterobacter* spp. (*n* = 53), *Pseudomonas aeruginosa* (*n* = 59), *Acinetobacter baumannii* (*n* = 14), and *Staphylococcus aureus* (*n* = 10). *Enterococcus faecium* was not isolated. These isolates had their identity confirmed by Matrix-Assisted Laser Desorption Ionization–Time of Flight (MALDI-ToF, Biotyper Compass version 4.1.100, BDAL library V13.0.0.2, Bruker Daltonics) as previously described (Assis et al., 2017; Brito et al., 2025). Subsequently, the isolates were subjected to antimicrobial susceptibility testing, as described in the following sections.

### 2.2 Antimicrobial susceptibility testing of *Salmonella* sp., *E. coli* and ESKAPE pathogens

The antimicrobial susceptibility test was performed by the agar disk diffusion method according to the Clinical and Laboratory Standards Institute (CLSI) VET01-Ed6 (CLSI, 2024) guidelines, and the inhibition zone diameters were interpreted following the CLSI M100-Ed31 (CLSI, 2021) criteria. The isolated bacteria were grown on BHI agar (Oxoid, Basingstoke, England), under aerobiosis, for 24-48 h at 37 °C. From their colonies, concentrations of 10^8^ viable cells (0.5 McFarland scale) were prepared using 3.5 mL of 0.85% buffered saline. Swabs from those dilutions were spread onto the surface of plates containing Muller Hilton agar (Oxoid, USA). The drug disks were distributed on the surface of the plates, which were incubated under aerobiosis, for 24 h at 37 °C. Then, the diameters of the inhibition zones were determined using a digital pachymeter. Critically important antimicrobials used in both human and veterinary medicine were selected for the antimicrobial susceptibility test. Isolates resistant to three or more antimicrobial classes were classified as multidrug-resistant (Magiorakos et al., 2012).

The antimicrobials chosen for each bacteria species were based on CLSI guidelines (CLSI, 2021; CLSI, 2024; CLSI VET01S, 2024). Regarding *Salmonella* spp., fourteen antimicrobials representing eleven different classes were tested: amikacin (30 μg), amoxicillin-clavulanic acid (30 μg), ampicillin (10 μg), azithromycin (15 μg), cephalothin (30 μg), ceftriaxone (30 μg), ciprofloxacin (5 μg), chloramphenicol (30 μg), gentamicin (10 μg), fosfomycin (200 μg), meropenem (10 μg), nitrofurantoin (300 μg), sulfamethoxazole-trimethoprim (1.25/23.75 μg), and tetracycline (30 μg).

For *E. coli*, *K. pneumoniae* and *Enterobacter* spp. isolates, thirteen antimicrobials representing eight different classes were tested: amoxicillin-clavulanic acid (30 μg), ampicillin (10 μg), cefotaxime (30 μg), ceftiofur (30 μg), cephalothin (30 μg), ciprofloxacin (5 μg), enrofloxacin (5 μg), ertapenem (10 μg), florfenicol (30 μg), gentamicin (10 μg), neomycin (30 μg), sulfamethoxazole-trimethoprim (1.25/23.75 μg), and tetracycline (30 μg). For *Salmonella* sp., *E. coli*, *K. pneumoniae* and *Enterobacter* spp. isolates, Extended-Spectrum β-Lactamase (ESBL) production was phenotypically assessed using the double-disk synergy test, in which a ceftriaxone disk (third-generation cephalosporin) was placed in proximity to an amoxicillin-clavulanate disk, and enhancement of the inhibition zone toward the clavulanate disk was interpreted as indicative of ESBL production (CLSI, 2021; Jarlier et al., 1988). In addition, isolates resistant to ceftriaxone were screened for AmpC β-lactamase production using a cefoxitin-cloxacillin double-disk synergy test (Edquist et al., 2013).

Antimicrobial selection for *A. baumannii* and *P. aeruginosa* was based on CLSI guidelines (CLSI, 2021; CLSI, 2024) and on their extensive intrinsic resistance profiles. Six antimicrobials were tested: aztreonam (30 μg), ceftazidime (30 μg), ciprofloxacin (5 μg), gentamicin (10 μg), imipenem (10 μg) and piperacillin-tazobactam (100/10 μg). *S. aureus* isolates were evaluated against eleven antimicrobials from different classes: cefoxitin (30 μg), penicillin (10 IU), ciprofloxacin (5 μg), chloramphenicol (30 μg), clindamycin (2 μg), erythromycin (15 μg), gentamicin (10 μg), nitrofurantoin (300 μg), rifampicin (5 μg), sulfamethoxazole-trimethoprim (1.25/23.75 μg), and tetracycline (30 μg). In all tests, *E. coli* ATCC® 25,922 and *S. aureus* ATCC® 25,923 were used as quality control strains.

### 2.3 Whole-genome sequencing and comparative genomics

Whole-genome sequencing was performed on selected isolates based on their antimicrobial resistance profiles. Priority was given to multidrug-resistant isolates belonging to bacterial groups included in the WHO Bacterial Priority Pathogens List 2024 (WHO, 2024), namely vancomycin-resistant *E. faecium*, methicillin-resistant *Staphylococcus aureus* (MRSA), carbapenem-resistant and/or extended-spectrum β-lactamase (ESBL)-producing Enterobacterales, and carbapenem-resistant *P. aeruginosa* and *A. baumannii*. To avoid overrepresentation of clonally related isolates, only one isolate from each bacterial species displaying a given antimicrobial resistance profile in the disk diffusion test was selected for whole-genome sequencing. This selection resulted in a total of 12 isolates, including five *E. coli*, three *K. pneumoniae*, and four *Salmonella* Minnesota strains.

For DNA extraction, each isolate was plated on brain heart infusion agar (BHI, Kasvi, Brazil) at 37 °C for 24 h. Genomic DNA was extracted using the Wizard Genomic DNA Purification Kit (Promega, EUA). Sequencing libraries were prepared with the Illumina DNA Prep Kit following the manufacture’s recommendations. Whole genome sequencing was performed on an Illumina MiSeq instrument using paired-end reads of 250 bp (2 × 250 bp). The raw data were analyzed using FastQC (Babraham Bioinformatics, England) and polished with Trimmomatic (Bolger et al., 2014) retaining only paired reads with a Phred quality of 30 or higher and a minimal size of 50 nucleotides (Zanon et al., 2026).

Genome assembly was performed using SPAdes v3.5.0 in careful mode (Bankevich et al., 2012; Nurk et al., 2013). Gap filling and polishing were performed using Pilon (Walker et al., 2014). The genomes were deposited in the National Center for Biotechnology Information (NCBI) under BioProject accession number PRJNA1337177.

ResFinder v4.7.2 (Zankari et al., 2017; Bortolaia et al., 2020) and PlasmidFinder v2.0.1 (Carattoli et al., 2014) were used with default parameters to identify acquired antimicrobial resistance determinants and conjugative plasmid replicons, respectively. Plasmid reconstruction was performed using MOB-recon tool v3.1.9 (Robertson and Nash, 2018) via the Galaxy Sciensano platform (Bogaerts et al., 2025)^1^, followed by BLASTn analysis against the NCBI database (Zhang et al., 2000). Multilocus sequence typing (MLST) was performed using the Pasteur scheme for *K. pneumoniae* and the Achtman scheme for *E. coli* and *S*. Minnesota, and sequence types (STs) were assigned using the PubMLST database (Jolley et al., 2018). SeqSero 1.2 was used for *Salmonella* serotype confirmation (Zhang et al., 2015).

For each bacterial species, the contigs were subjected to single nucleotide polymorphism (SNP) analysis using CSIPhylogeny (Price et al., 2010; Kaas et al., 2014) with a minimal Z-score of 1.96 and a minimum sequencing depth of 10 × at SNP positions. *S*. Minnesota ATCC 49284 strain (BioSample accession number SAMN01823721), *Escherichia coli* strain 190693 (BioSample accession number SAMN14137859) and *Klebsiella pneumoniae* strain CPKp1825 (BioSample accession number SAMN12061308) were used as references. Strains of *S*. Minnesota assigned to ST548, originating from poultry (*n* = 38) and humans (*n* = 21), were obtained from the Bacterial and Viral Bioinformatics Resource Center (BV-BRC) database (Olson et al., 2023) and used for comparison (Supplementary File 1). Additionally, ST38 (*E. coli*) and ST307 (*K. pneumoniae*) strains were selected from major importers of Brazilian chicken meat (China, Japan, United Arab Emirates, Saudi Arabia, and South Africa), along with strains from Brazil (Supplementary File 1). From each alignment generated we performed a recombination filtering using the Gubbins tool (Croucher et al., 2015) with the settings min_snp = 3 and *p*-value = 0.05. The filtered alignments were then used to generate the phylogenetic tree with the IQTREE 3 software (Wang et al., 2026) with the bootstrap set for 1000. The trees were visualized using iTOL v.6 online (Letunic and Bork, 2019) and midpoint rooted. All strains were retrieved from the same database. The phylogenetic trees were visualized using iTOL v.6 online (Letunic and Bork, 2019) and midpoint rooted.

### 2.4 Statistical analysis

Antimicrobial susceptibility data, including resistance to different classes of drugs and multidrug resistance profiles, were summarized using frequency tables. Univariate analysis was conducted using Fisher’s exact test to assess associations and calculate odds ratios (OR) between resistance to specific antimicrobials and multidrug resistance. All analyses were performed using Stata/SE 18.0 (StataCorp, 2023).

## 3 Results

### 3.1 *Salmonella enterica* sorovar Minnesota

#### 3.1.1 Antimicrobial susceptibility

A total of 25 isolates out of the 48 tested (52.1%) were resistant to at least one antimicrobial, and all of them were classified as multidrug-resistant. More than half of the isolates were resistant to folate pathway antagonist (60.4%), first-generation cephalosporin (54.2%), third-generation cephalosporin (52.1%), tetracyclines (52.1%) and penicillins (52.1%). In addition, more than a quarter of the isolates (14/48 = 29.2%) were resistant to fluoroquinolones (Figure 1). Phenotypic testing revealed that 25 strains (25/48 = 52.1%) were ESBL producers, and the same number was positive for AmpC. Notably, 24 of these (24/25 = 96%) were identified as the Minnesota serotype. A strong association was observed between serovar Minnesota and the multidrug-resistant phenotype (OR, 767.7; 95% CI, 29.8–1,804.6; *p <* 0.0001). Furthermore, isolates resistant to folate pathway antagonists, first and third-generation cephalosporin, fluoroquinolones, penicillins and tetracyclines were more likely to exhibit a multidrug-resistant profile (*p <* 0.05).

**Figure 1:**
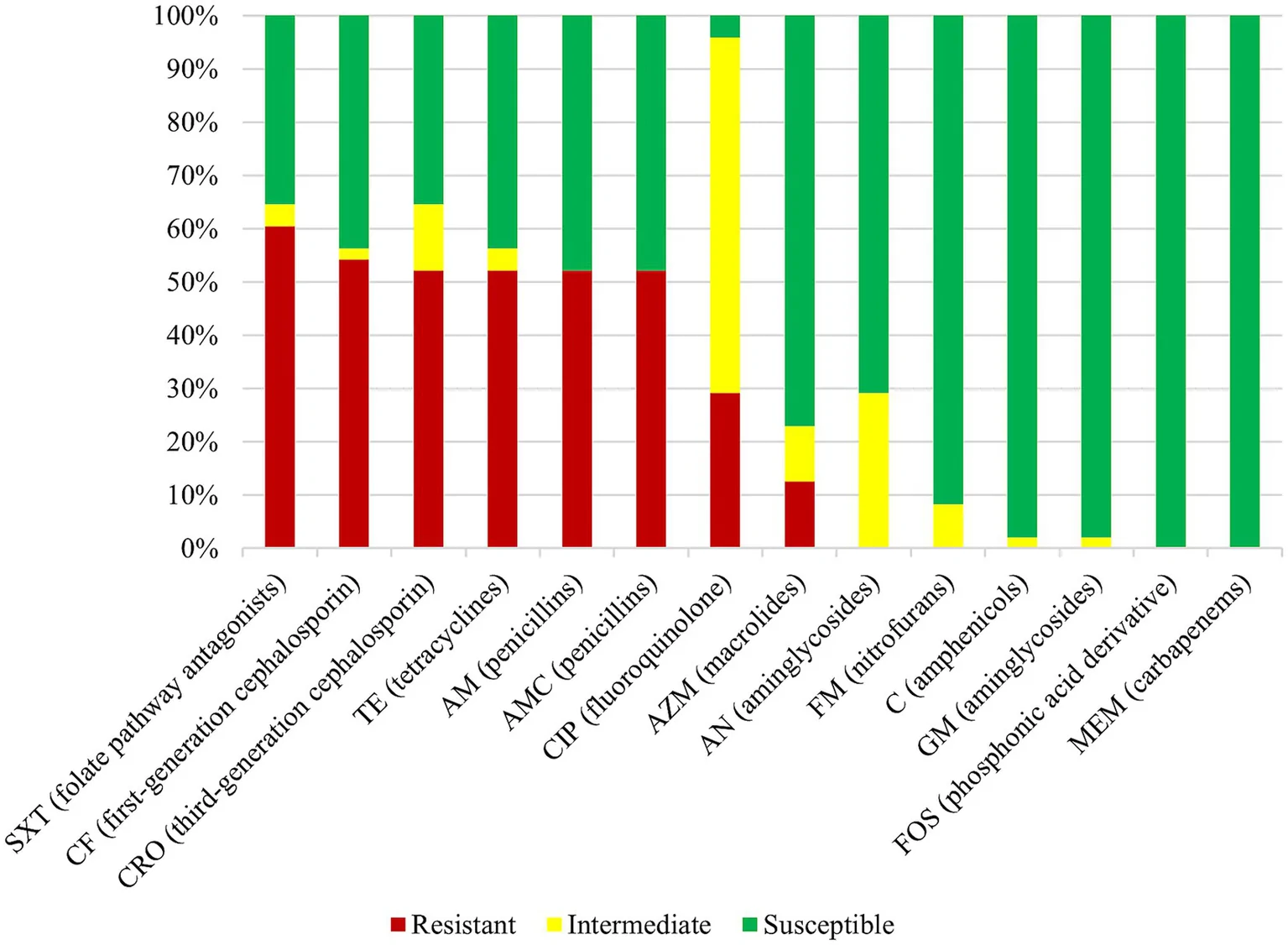
Antimicrobial resistance profile of *Salmonella* sp. isolates (*n* = 48) from chicken carcasses. STX, sulfamethoxazole-trimethoprim (1.25/23.75 μg); CF, cephalothin (30 μg); CRO, ceftriaxone (30 μg); TE, tetracycline (30 μg); AM, ampicillin (10 μg); AMC, amoxicillin-clavulanate (30 μg); CIP, ciprofloxacin (5 μg); AZM, azithromycin (15 μg); AN, amikacin (30 μg); FM, nitrofurantoin (300 μg); C, chloramphenicol (30 μg); GM, gentamicin (10 μg); FOS, fosfomycin (200 μg); MEM, meropenem (10 μg).

#### 3.1.2 Antimicrobial resistance determinants and plasmid replicons

The 12 sequenced isolates (four *Salmonella* Minnesota, five *E. coli* and three *K. pneumoniae*) presented genome coverages ranging from 83 × to 133 × and genome sizes between 4.6 and 5.7 Mbp. Additional details regarding sequencing and assembly quality are provided in Supplementary File 5. All sequenced strains harbored antimicrobial resistance genes associated with resistance to aminoglycosides (*aac(6′)-Iaa*, *aadA1*, *aph(3′)-Ia*), AmpC β-lactamase (*bla_CMY-2_*), folate pathway antagonists (*sul2*), and tetracyclines (*tet(A)*) (Supplementary material 2). In addition, all strains carried the plasmid-mediated quinolone resistance (PMQR) gene *qnrB19* (Figure 2). The resistance genes identified were highly consistent with the phenotypic antimicrobial susceptibility profiles, demonstrating a strong concordance between genotypic predictions and phenotypic resistance patterns.

**Figure 2:**
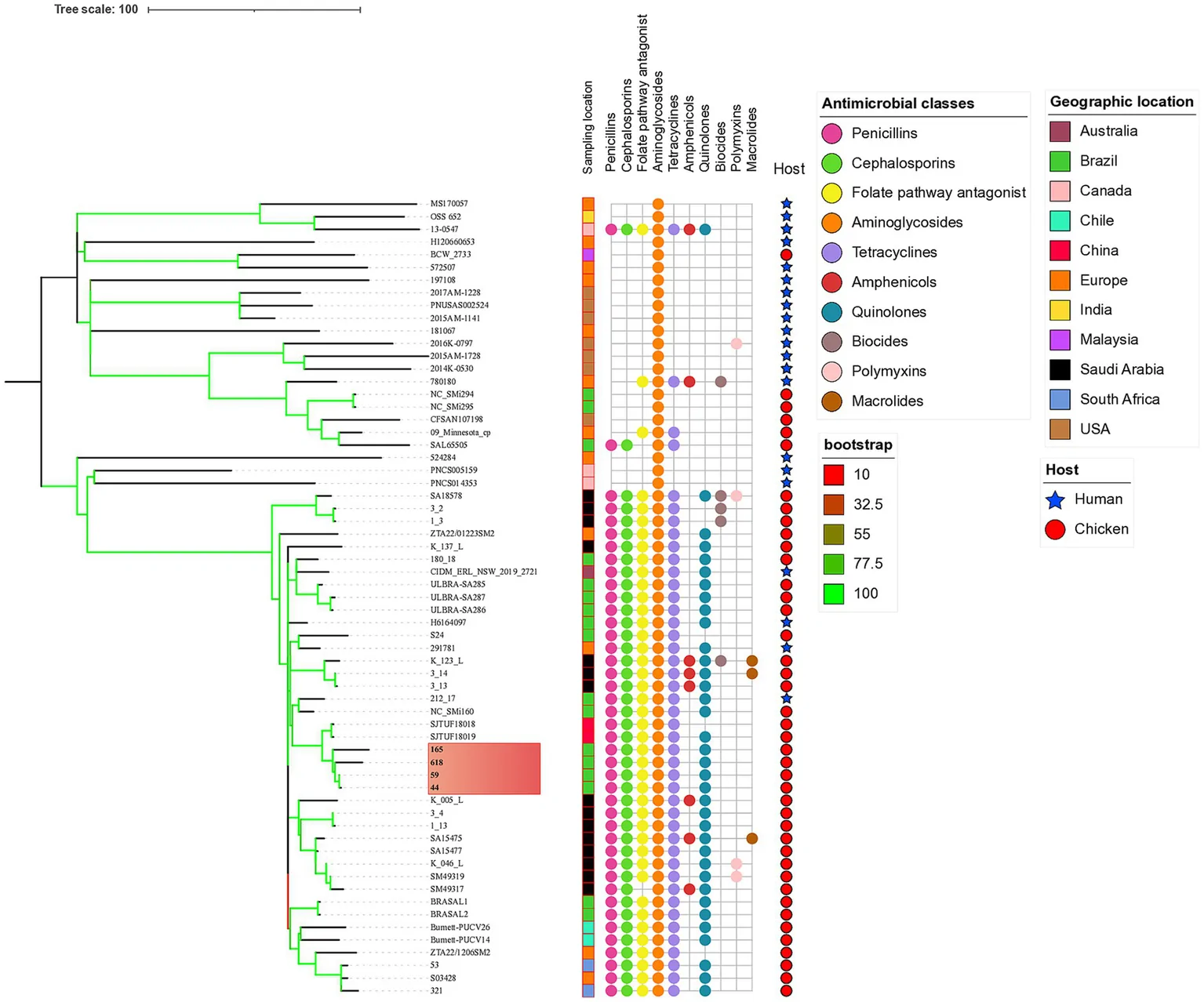
Genomic single-nucleotide polymorphism (SNP) analysis showing the genetic relationships among *S. Minnesota* isolates from chicken and human sources. Strains from the present study are highlighted in bold and marked in red (strains 44, 59, 165, and 618). *S. Minnesota* ST548 isolates from poultry and human sources were retrieved from the BV-BRC database and included for comparison. The phylogenetic tree was visualized using iTOL (Letunic and Bork, 2019) online, with midpoint rooting.

Plasmids Col440I and IncA/C2 were detected in all *S*. Minnesota isolates from the present study, with *qnrB19* located on Col440I and not associated with other resistance genes (Supplementary material 3).

#### 3.1.3 Sequence typing and SNP-based phylogenetic analysis

Multilocus sequence typing (MLST) analysis demonstrated that all isolates belonged to sequence type ST548. SNP-based analysis revealed a high similarity among the strains recovered in the present study (Figure 2), with pairwise differences ranging from 1 to 31 SNPs. When compared with publicly available genomes, the isolates from the present study differed by up to 320 SNPs (Supplementary material 4). The closest relationship was observed for strain 59, which differed by 31 SNPs from two chicken meat isolates from China, (SJTUF18018 and SJTUF18019). Notably, the isolates also showed close genetic relatedness to human-derived strains. The nearest human-associated genome was a Brazilian isolate (H6164097), which differed from isolate 59 by only 33 SNPs and by no more than 44 SNPs across the entire dataset. Additionally, three other human-derived strains were also closely related to our isolates, including another Brazilian strain (212_17), with a minimum difference of 37 SNPs, as well as strains from Australia (CIDM_ERL_NSW_2019_2721) and the United Kingdom (291781), differing by a minimum of 51 and 37 SNPs, respectively.

### 3.2 Escherichia coli

#### 3.2.1 Antimicrobial susceptibility

A total of 98% (97/99) of the isolates were resistant to at least one antimicrobial, and 84.8% (84/99) were classified as multidrug-resistant. Furthermore, 56 isolates (56/99 = 56.6%) were confirmed as ESBL producers. High resistance rates were observed for penicillins (82.8%), first-generation cephalosporins (75.8%), and aminoglycosides (71.7%). Furthermore, more than half of the isolates were resistant to folate pathway antagonists (56.6%), third-generation cephalosporins (56.6%), and tetracyclines (55.6%) (Figure 3).

**Figure 3:**
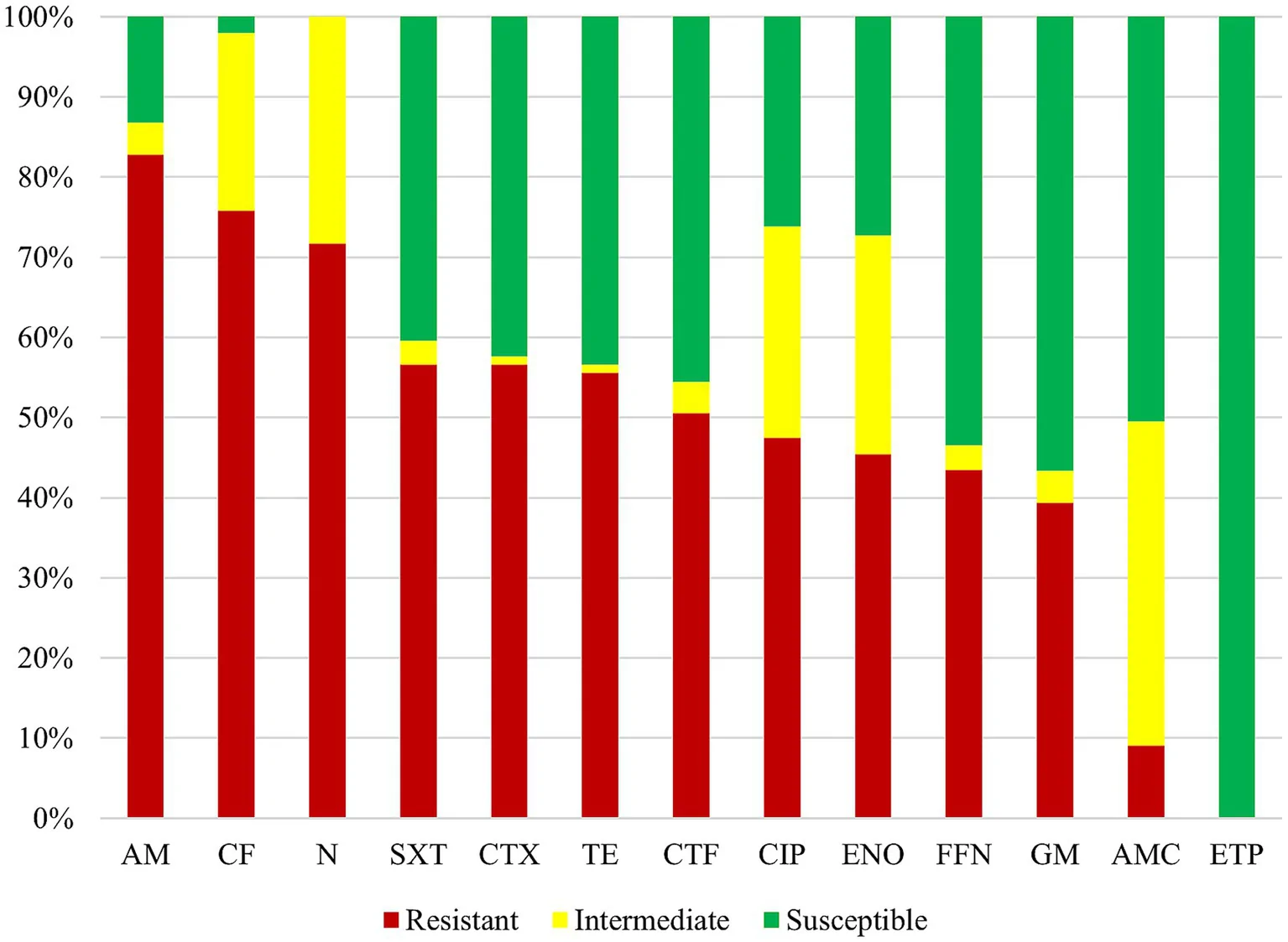
Antimicrobial resistance profile of *Escherichia coli* isolates (*n* = 99) recovered from chicken carcasses. AM, ampicillin (10 μg); CF, cephalothin (30 μg); N, neomycin (30 μg); STX, sulfamethoxazole-trimethoprim (1.25/23.75 μg); CTX, cefotaxime (30 μg); TE, tetracycline (30 μg); CTF, ceftiofur (30 μg); CIP, ciprofloxacin (5 μg); ENO, enrofloxacin (5 μg); FFN, florfenicol (30 μg); GM, gentamicin (10 μg); AMC, amoxicillin-clavulanate (30 μg); ETP, ertapenem (10 μg).

Statistical analysis revealed that resistance to penicillins, first- and third-generation cephalosporin, folate pathway antagonists, tetracyclines, amphenicols, and fluoroquinolones was significant associated with the multidrug-resistant phenotype (*p <* 0.05).

#### 3.2.2 Antimicrobial resistance determinants and plasmid replicons

All isolates harbored resistance genes associated with aminoglycosides and folate pathway antagonists (Supplementary material 2). Additionally, point mutations in *gyrA* (p. S83L) and *parC* (p. S80I), associated with fluoroquinolone resistance, were identified in all isolates except one, which carried the PMQR gene *qnrB19*. Three isolates (26, 215, and 574) harbored ESBL-associated genes, including *bla_CTX-M-55_* in two isolates and *bla_CTX-M-65_* in one. The AmpC β-lactamase gene *bla_CMY-2_* was detected in isolate 9.

Thirteen different plasmid replicons were detected among the sequenced strains. Isolate 9 stood out due to the presence of an IncB/O/K/Z plasmid carrying the *bla_CMY-2_* gene, as well as an IncQ1 plasmid harboring resistance genes associated with aminoglycosides (*aph(3′)-Ia*) and phosphonic acid derivatives (*fosL1*) (Supplementary material 3).

#### 3.2.3 Sequence typing and SNP-based phylogenetic analysis

Five different sequence types were detected among the sequenced *E. coli* strains, including ST38, ST1485, ST295, ST351 and a novel ST: 18519. SNP-based phylogenetic analysis was conducted using strain 9 (ST38, clonal complex [CC] 38), a high-risk global clone. SNP-based analysis revealed substantial divergence between our strain and isolates from Brazil and major importers countries of Brazilian chicken meat (Supplementary material 4). Only one isolate, originating from an avian host in Brazil, clustered with the strain from the present study, differing by 90 SNPs (Figure 4).

**Figure 4:**
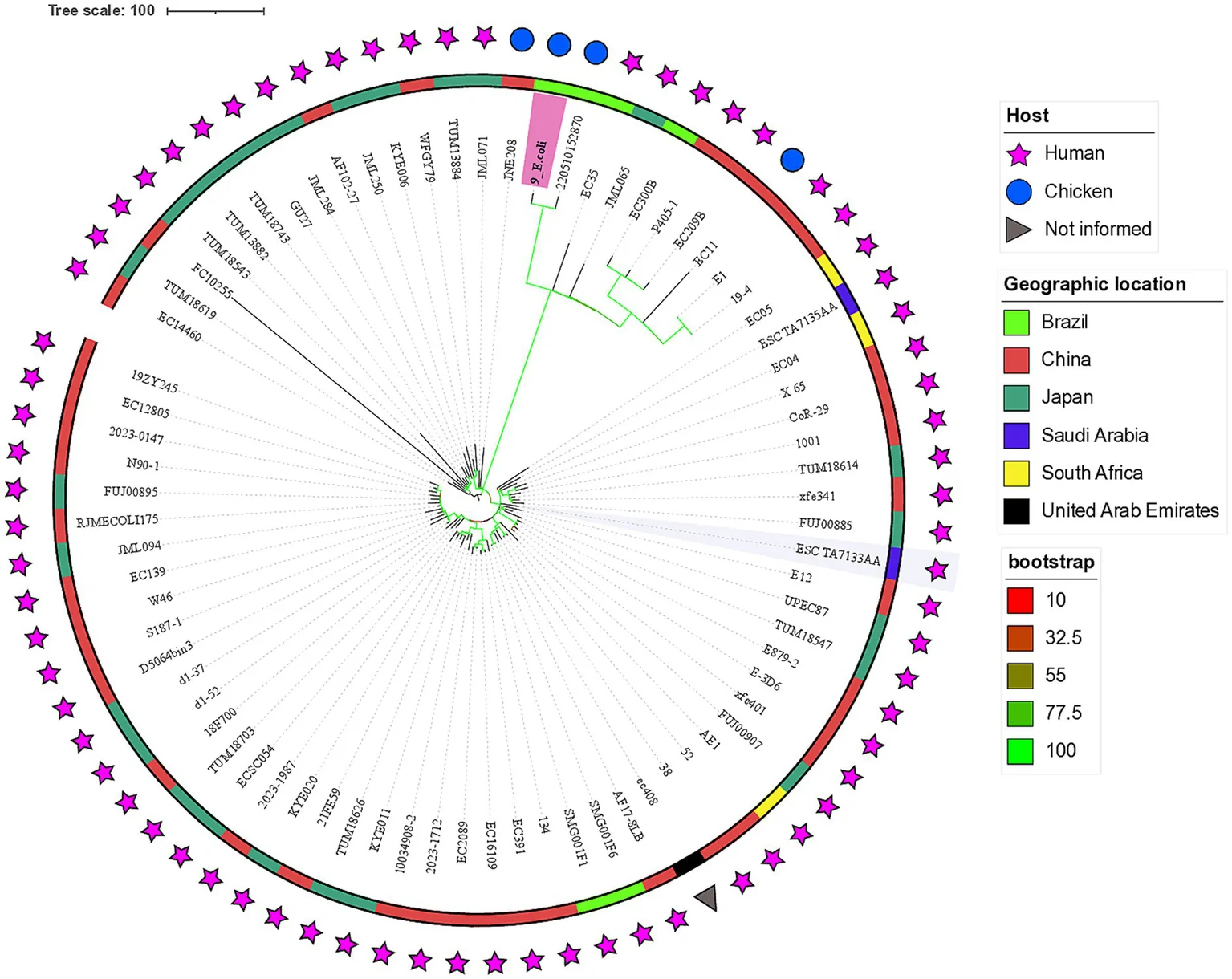
Single-nucleotide polymorphism (SNP) analysis showing the genetic relationships among *E. coli* ST38 isolates from chicken and human sources. The strain from the present study is highlighted in bold and marked in pink (strain 9). *E. coli* ST38 isolates from Brazil and major importers of Brazilian chicken meat were retrieved from the BV-BRC database and included for comparison. The phylogenetic tree was visualized using iTOL (Letunic and Bork, 2019) online, with midpoint rooting.

### 3.3 ESKAPE

#### 3.3.1 Klebsiella pneumoniae

##### 3.3.1.1 Antimicrobial susceptibility

Among *K. pneumoniae* isolates, all strains were resistant to at least one antimicrobial, and 47.1% (32/68) were classified as multidrug-resistant. In addition, 13.2% of the isolates (9/68) were confirmed as ESBL producers. High resistance rates were observed for penicillins (98.5%), fluoroquinolones (58.8%), and aminoglycosides (55.9%). Furthermore, more than a quarter of the isolates were resistant to tetracyclines (27.9%) (Figure 5). In the present study, isolates resistant to first and third-generation cephalosporin, folate pathway antagonists, tetracyclines, amphenicols, and aminoglycosides were more likely to exhibit a multidrug-resistant profile (*p <* 0.05). Additionally, fluoroquinolone resistance was strongly associated with a multidrug-resistant phenotype (OR, 13; 95% CI, 4.1–41.7; *p <* 0.0001).

**Figure 5:**
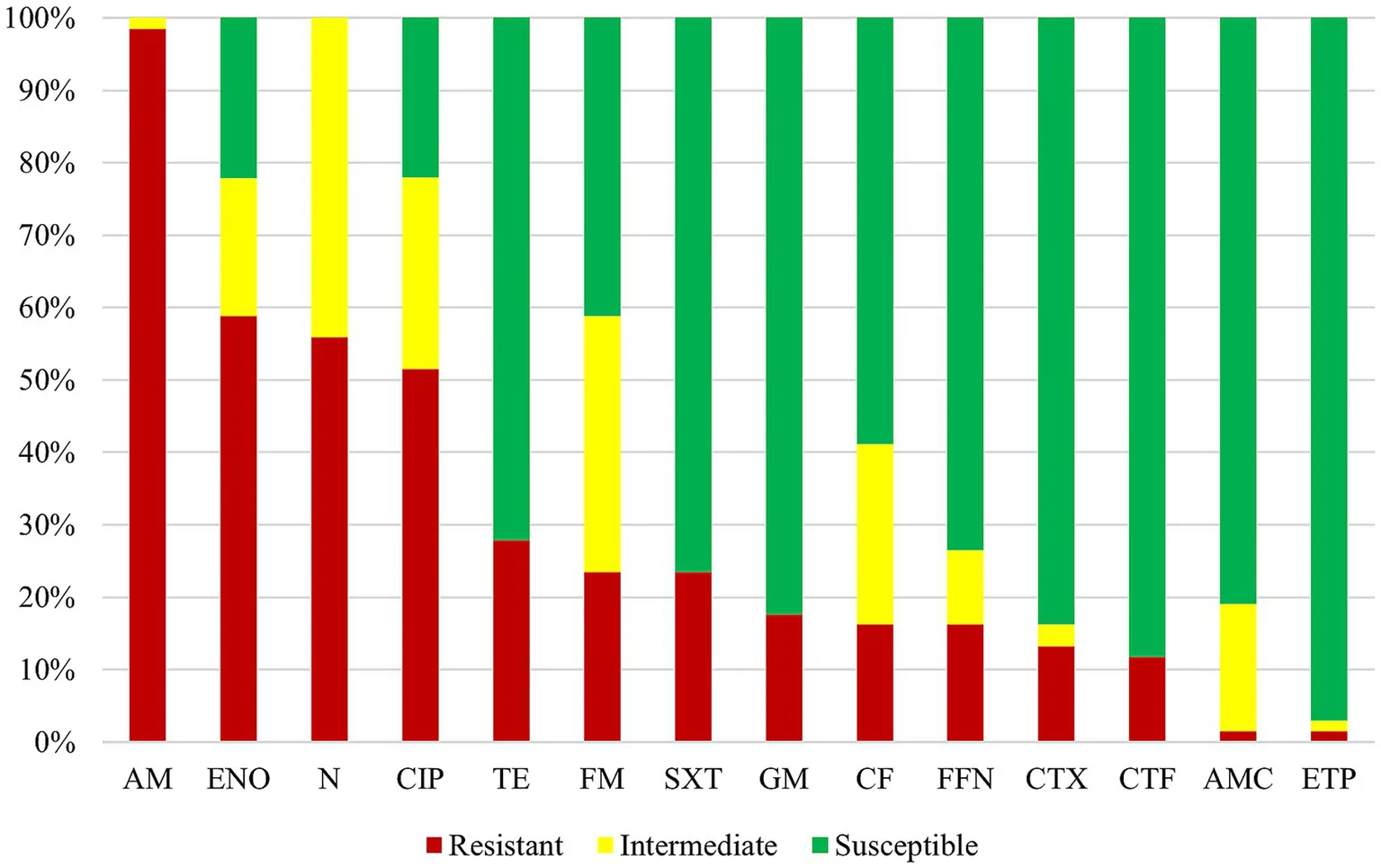
Antimicrobial resistance profile of *Klebsiella pneumoniae* isolates (*n* = 68) from chicken carcasses. AM, ampicillin (10 μg); CF, cephalothin (30 μg); N, neomycin (30 μg); STX, sulfamethoxazole-trimethoprim (1.25/23.75 μg); CTX, cefotaxime (30 μg); TE, tetracycline (30 μg); CTF, ceftiofur (30 μg); CIP, ciprofloxacin (5 μg); ENO, enrofloxacin (5 μg); FFN, florfenicol (30 μg); GM, gentamicin (10 μg); AMC, amoxicillin-clavulanate (30 μg); ETP, ertapenem (10 μg).

##### 3.3.1.2 Antimicrobial resistance determinants and plasmid replicons

All *K. pneumoniae* isolates sequenced in the present study harbored antimicrobial resistance genes associated with multiple antimicrobials classes, including aminoglycosides, beta-lactams, amphenicols, folate pathway antagonists, macrolides, phosphonic acid derivatives, and tetracyclines (Supplementary material 2). Additionally, all isolates harbored *oqxA* and *oqxB*, which encode a multidrug efflux pump that contributes to resistance against quinolones, folate pathway antagonists, and amphenicols. Isolates 54 and 569 stood out due to the presence of resistance genes associated with rifampicin (*ARR-3*) and biocides (*formA* and *qacH*), respectively.

Notably, all *K. pneumoniae* strains harbored β-lactamase-encoding genes, including several *bla_SHV_* variants and *bla_CTX-M-15_*. The *bla_SHV_* variants identified were *bla_SHV-11_* and *bla_SHV-67_* (isolate 54), *bla_SHV-193_*, *bla_SHV-178_*, *bla_SHV-80_* and *bla_SHV-36_* (isolate 569), *bla_SHV-106_* and *bla_SHV-28_* (isolate 636). Among these, *bla_SHV-106_* and *bla_CTX-M-15_* are ESBL-encoding genes. Furthermore, point mutations associated with reduced susceptibility to fluoroquinolones, cephalosporins and carbapenems were identified in all isolates from the present study (Supplementary material 2).

##### 3.3.1.3 Sequence typing and SNP-based phylogenetic analysis

Three distinct sequence types were detected among the sequenced *K. pneumoniae* strains, including ST4536, ST323 and ST307. Strain 636, belonging to the globally disseminated high-risk clone ST307 (CC307), was selected for comparative phylogenomic analysis. Comparative genomes were primarily selected from human isolates originating from major importers of Brazilian poultry meat, aiming to investigate potential epidemiological links. In addition, due to the limited availability of poultry-associated ST307 strain, the only three available chicken-derived genomes were included, all originating from Portugal. SNP-based phylogenomic analysis revealed considerable genetic divergence between strain 636 and the comparative isolates, with pairwise SNP distances ranging from 60 to 132 SNPs (Supplementary material 4) (Figure 6).

**Figure 6:**
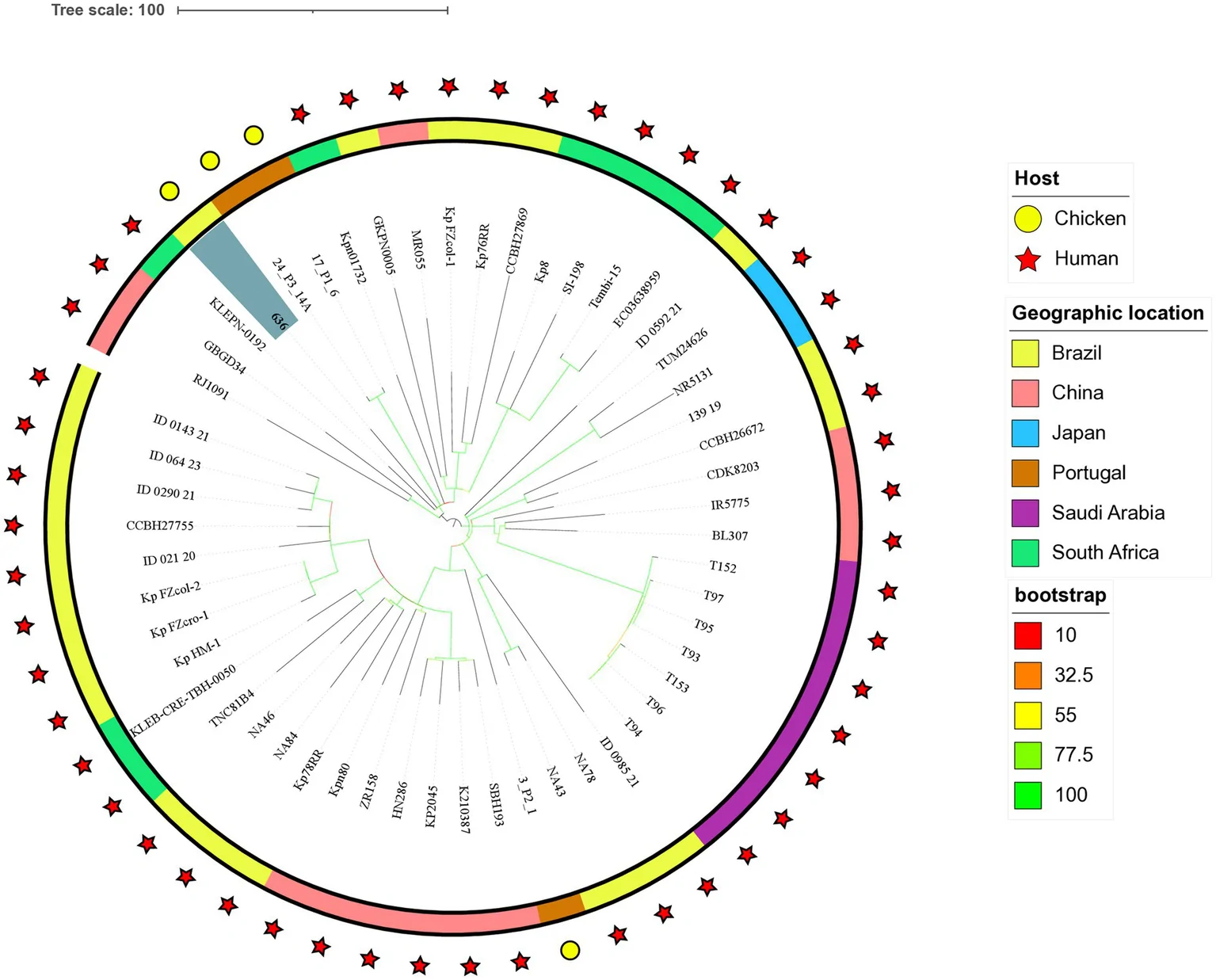
Single-nucleotide polymorphism (SNP) analysis showing the genetic relationships among *K. pneumoniae* ST307 isolates from chicken and human sources. The strain from the present study is highlighted in bold and marked in blue (strain 636). *K. pneumoniae* ST307 isolates from Brazil and major importers of Brazilian chicken meat were retrieved from the BV-BRC database and included for comparison, along with all publicly available chicken-derived ST307 genomes (*n* = 3). The phylogenetic tree was visualized using iTOL (Letunic and Bork, 2019) online, with midpoint rooting.

#### 3.3.2 Enterobacter spp., *Pseudomonas aeruginosa*, *Acinetobacter baumannii* and *Staphylococcus aureus*

All *Enterobacter* strains, belonging to the *E. cloacae* complex (Paauw et al., 2008), were resistant to at least one antimicrobial, and 83% (44/53) were classified as multidrug-resistant. Among the *Pseudomonas aeruginosa* isolates, eight strains exhibited resistance to at least one antimicrobial, although none were multidrug-resistant. Six *Acinetobacter baumannii* isolates showed resistance to at least one antimicrobial, including two multidrug-resistant strains. For *Staphylococcus aureus*, five isolates were classified as multidrug-resistant, all exhibiting inducible clindamycin resistance (D-test positive), while no methicillin-resistant *S. aureus* (MRSA) isolates were identified.

## 4 Discussion

Assessing the microbiological status of broiler chickens during the slaughtering process is essential to minimize public health risks and ensure meat quality (Wyink et al., 2025). This study aimed to characterize the antimicrobial resistance profiles of *Salmonella* spp., *E. coli*, and ESKAPE pathogens isolated from chicken carcasses through phenotypic antimicrobial susceptibility testing and comparative genomic analyses of selected multidrug-resistant isolates. Among the *Salmonella* serotypes identified, a high frequency of Minnesota was observed, accounting for the majority of the resistance profiles detected. In addition, clinically relevant sequence types among *E. coli* and *K. pneumoniae* were identified, reinforcing the role of the poultry production chain as a potential reservoir and foodborne source of multidrug-resistant pathogens.

Salmonellosis remains a major concern in poultry production, as food products of poultry origin are recognized as one of the principal sources of human infection (Wang et al., 2023; Logue et al., 2024). In this study, *S.* Minnesota was the predominant serovar, comprising 50% of the isolates, in agreement with previous studies highlighting the importance and emergence of this serovar in the Brazilian poultry production chain (Voss-Rech et al., 2015; Mendonça et al., 2019; Monte et al., 2019; Kipper et al., 2020). Furthermore, MLST analysis demonstrated that all isolates belonged to sequence type ST548, a lineage previously described and strongly associated with the *Salmonella enterica* serovar Minnesota (Monte et al., 2019; Raghuram et al., 2026). Currently, the Brazilian Ministry of Agriculture and Livestock requires poultry slaughterhouses to monitor *Salmonella* in chicken carcasses, but surveillance is limited to five serovars: *S. enteritidis*, *S. typhimurium*, *S. gallinarum*, *S.* Pullorum, and monophasic *S. typhimurium* (Ministério da Agricultura, Pecuária e Abastecimento, 2016). Consequently, no specific regulations address *S.* Minnesota, despite its emergence in the Brazilian poultry industry and its potential public health relevance (Alikhan et al., 2022; Mesa et al., 2026).

In addition to its high prevalence in poultry supply chains, *S.* Minnesota plays a key role in the dissemination of antimicrobial resistance, with multidrug resistance commonly observed among its clonal lineages (Campos et al., 2018; Silveira et al., 2021; Huang et al., 2025; Mesa et al., 2026). In fact, in the present study, among the 25 multidrug-resistant strains, 24 (96%) belonged to serovar Minnesota, with all isolates confirmed as ESBL producers and positive for AmpC production. Furthermore, the four sequenced isolates harbored multiple antimicrobial resistance determinants, including the AmpC β-lactamase gene *bla_CMY-2_* and the PMQR gene *qnrB19*, which are commonly associated with *S.* Minnesota and confer resistance to broad-spectrum β-lactams, including third-generation cephalosporins and fluoroquinolones, respectively (Monte et al., 2019; Saidenberg et al., 2023). Interestingly, in all our isolates these genes were associated with IncA/C2 and Col440I plasmid replicons, which are commonly known to harbor antimicrobial resistance determinants such as the *bla_CMY-2_*/*sul2*/*tet(A)* complex and *qnrB19*, respectively (Dos Santos et al., 2024).

In comparison with other *S.* Minnesota strains from previous studies, our isolates clustered with strains of chicken origin from China. Notably, that country is one of the main importers of Brazilian poultry meat, and a recent study demonstrated the introduction of multidrug-resistant *S.* Minnesota strains from Brazil into China via imported chicken meat (ABPA, 2025; Li et al., 2025). Considering Brazil’s position as one of the world’s largest poultry exporters, international trade may contribute to the global dissemination of successful *S*. Minnesota lineages and their associated antimicrobial resistance determinants (Raghuram et al., 2026). The phylogenetic analysis also showed that the *S*. Minnesota strains were closely related to isolates recovered from human cases in Brazil, the United Kingdom, and Australia. Although *S*. Minnesota has been less frequently associated with human disease, several studies highlight its potential risk to public health, particularly due to the diverse range of antimicrobial resistance determinants harbored by this serovar (Alikhan et al., 2022; Moreira et al., 2022; Dos Santos et al., 2024; Huang et al., 2025; Raghuram et al., 2026). Beyond the risk to humans, recent evidence highlights the risk of *S*. Minnesota to companion animals, reinforcing its potential for transmission to pets, especially through raw meat–based diets (Sartori et al., 2025).

ESBL-producing *E. coli* plays a significant role, being classified as a critical priority pathogen by the WHO Bacterial Priority Pathogens List (WHO, 2024). In this study, 56.6% of the *E. coli* isolates from chicken carcasses were classified as ESBL-producing, representing a higher frequency compared to similar studies with chicken carcass in Africa and Asia (Chishimba et al., 2016; Eltai et al., 2020; Abubakar et al., 2023; Kabir et al., 2025; Telli et al., 2025). Moreover, ESBL genes belonging to the CTX-M group were the most frequently detected, being identified in three out of the five sequenced isolates. The AmpC β-lactamase gene *bla*_CMY-2_ was also detected in one isolate in this study. Noteworthy, CMY-2-producing *E. coli* is considered the predominant plasmid-mediated AmpC (pAmpC). Taking together, the presence of *bla*_CTX-M_ and *bla*_CMY-2_ represents a public health concern given its potential dissemination through the food chain through contaminated meat (Mo et al., 2017; Daehre et al., 2018; Pietsch et al., 2018; Kaesbohrer et al., 2019; Che et al., 2023).

MLST analysis revealed that one strain belonged to ST38, a sequence type commonly identified among *E. coli* isolates from poultry and frequently associated with the AmpC β-lactamase gene *bla*_CMY-2_, as observed in this study (Mo et al., 2016; Myrenås et al., 2018; Päivärinta et al., 2020). *E. coli* ST38 represents an emerging clone, with high levels of multidrug resistance (Roer et al., 2026). In addition, this lineage is linked to hospital and community-associated outbreaks in humans (Hammerum et al., 2020; Lindemann et al., 2023). Recent studies have described the existence of two distinct clusters among ST38 *E. coli* isolates, separating strains of poultry and human origin (Mo et al., 2023; Roer et al., 2026). In agreement with these findings, phylogenetic analysis in the present study revealed a clear divergence between our isolate and those from human sources, with clustering observed only with a single chicken-derived strain from Brazil. Despite this finding, the zoonotic potential should not be neglected, as a large proportion of isolates causing human infections may originate from food sources (Abgottspon et al., 2014; Pietsch et al., 2018; Díaz-Jiménez et al., 2020; Roy Chowdhury et al., 2023). Furthermore, recent studies have identified genomic similarities between poultry-associated *E. coli* and strains responsible for urinary tract infections in humans, supporting the role of poultry products as potential reservoirs of antimicrobial-resistant *E. coli* (Aziz et al., 2024; Wang et al., 2026).

ESKAPE pathogens are among the leading causes of antimicrobial resistance related deaths worldwide, being associated with healthcare-associated infections (Zhen et al., 2019; De Oliveira et al., 2020). In this study, all members of the ESKAPE group were detected, except for *E. faecium*. Among these, ESBL-producing *K. pneumoniae* were identified in 13.2% of the isolates. Additionally, all three sequenced isolates harbored ESBL genes on plasmid replicons. Among these, IncFIB (pNDM-Mar), a well-known reservoir of *bla*_NDM-1_ conferring resistance to carbapenems, was identified in two strains (54 and 569) (Villa et al., 2012; Stein et al., 2023; Loconsole et al., 2024; Rigatou et al., 2025). In contrast, a multi-replicon plasmid carrying IncFIB(K)/IncFII(K) was detected in one strain (636), associated with ESBL genes. Such multi-replicon plasmids are commonly linked to the worldwide dissemination of ESBLs and carbapenemases in *K. pneumoniae* clinical isolates (Bi et al., 2018; Zhang et al., 2023; Ma et al., 2024). Noteworthy, isolate 636 was assigned to ST307 in this study, a globally disseminated multidrug-resistant clone of *K. pneumoniae* (Wyres et al., 2020; Budia-Silva et al., 2024). Among the strains included for comparison, our isolate showed marked divergence, in agreement with previous studies (Mourão et al., 2023, 2024). Nevertheless, the identification of ST307 in chicken carcasses is of particular concern give its well-stablished clinical relevance (Long et al., 2017; Villa et al., 2017). Collectively, these findings highlight the importance of chicken meat as a potential source of ESBL-producing *K. pneumoniae* for humans, including internationally disseminated high-risk clones, and highlight the potential for foodborne transmission of clinically relevant strains (Projahn et al., 2019; Rodrigues et al., 2022). Furthermore, the association of these resistance genes with mobile genetic elements, particularly plasmids, may facilitate horizontal gene transfer and contribute to their dissemination among bacterial populations (Tang et al., 2014).

Among Enterobacteriales isolates in the present study, it is noteworthy that none of the *E. coli* and *K. pneumoniae* isolates were susceptible to neomycin. The high frequency of non-susceptibility suggests that neomycin would have limited activity against the bacterial population evaluated in this study, which corroborates previous studies in broiler chicken (Roth et al., 2019; Oliveira et al., 2022). This finding should, however, be interpreted in light of the CLSI VET01S breakpoints used in the present study, as the broad intermediate range for neomycin (16–24 mm) may have contributed to the absence of susceptible isolates.

The present study has some limitations. First, sampling was restricted to a single federally inspected poultry slaughterhouse, from which only 60 chicken carcasses were collected. Although this facility supplies both the domestic and international markets, allowing an initial assessment of the potential public health risk associated with poultry products intended for export, the findings cannot be generalized to the entire Brazilian poultry industry. Therefore, the present study should be regarded as a preliminary screening of antimicrobial resistance in this production setting. Another limitation regarding the sampling is that multiple bacterial isolates could be recovered from the same carcass, while statistical analyses were conducted at the isolate level without accounting for within-carcass clustering. Consequently, isolates originating from the same carcass may not represent fully independent observations and could potentially be clonally related. This may have affected the magnitude and precision of the estimated associations, and these results should therefore be interpreted with caution. Second, plasmid reconstruction was based exclusively on short-read sequencing data. It is well recognized that short-read assemblies cannot always resolve complete plasmid structures (Arredondo-Alonso et al., 2017). Thus, the exact genetic context of the antimicrobial resistance determinants should be interpreted with caution. Nevertheless, our findings are consistent with recent reports identifying chicken carcasses as a potential reservoir of clinically relevant antimicrobial-resistant bacteria associated with human infections. Further studies involving a larger number of slaughterhouses across different Brazilian regions are needed to better understand the national epidemiology of these pathogens. In addition, broader comparative genomic analyses including isolates from both Brazilian human infections and countries importing Brazilian chicken meat would provide valuable insights into the potential dissemination of high-risk bacterial lineages through the food chain.

## 5 Conclusion

This study demonstrated the occurrence of multidrug-resistant *Salmonella*, *E. coli*, and ESKAPE pathogens carrying clinically relevant antimicrobial resistance genes in chicken carcasses. The predominance of *S.* Minnesota harboring important resistance determinants, together with the detection of internationally disseminated high-risk clones such as *E. coli* ST38 and *K. pneumoniae* ST307, suggests that poultry products may serve as reservoirs and potential vehicles for the dissemination of antimicrobial-resistant bacteria and antimicrobial resistance genes.

## Data Availability

All relevant data is contained within the article. The original contributions presented in the study are included in the article/ Supplementary Material, further inquiries can be directed to the corresponding authors.

## Footnotes

1
https://galaxy.sciensano.be
