# Supplementary material for: Antimicrobial resistance and genomic characterization of Salmonella Minnesota, ESBL-producing Escherichia coli, and ESKAPE pathogens isolated from Brazilian broiler chicken carcasses
Source: Front Microbiol. 2026 Sep 15;17:1917698. doi: 10.3389/fmicb.2026.1917698 (PMC13619903; doi:10.3389/fmicb.2026.1917698)
Supplement: Supplementary file 1 [file Table_2.DOCX]

Supplementary Material 2

**Table S2**. Genomic characterization of the four *S.* Minnesota isolates based on whole-genome sequencing.

| **Strain ID** | **Sequence type (ST)** | **Resistance genotype** | **Plasmid replicon** |
| --- | --- | --- | --- |
| 44 | 548 | *aac(6')-Iaa*, *aadA1*, *aph(3')-Ia*, *qnrB19*, *bla_cmy-2_*, *sul2*, *tet(A)* | Col440I, IncA/C2 |
| 59 | 548 | *aac(6')-Iaa*, *aadA1*, *aph(3')-Ia*, *qnrB19*, *bla_cmy-2_*, *sul2*, *tet(A)* | Col440I, IncA/C2 |
| 165 | 548 | *aac(6')-Iaa*, *aadA1*, *aph(3')-Ia*, *qnrB19*, *bla_cmy-2_*, *sul2*, *tet(A)* | Col440I, IncA/C2 |
| 618 | 548 | *aac(6')-Iaa*, *aadA1*, *aph(3')-Ia*, *qnrB19*, *bla_cmy-2_*, *sul2*, *tet(A)* | Col440I, IncA/C2 |

**Table S2**. Genomic characterization of the five *E. coli* isolates based on whole-genome sequencing.

| **Strain ID (ST)** | **Resistance genotype** | **Mutations** | **Plasmid replicon** |
| --- | --- | --- | --- |
| 9 (ST38; CC38) | *aac(3)-VIa, aadA1, aph(3')-Ia, aph(4)-Ia, aac(3)-IV, blaCMY-2, fosL1, sul1, sul2, tet(B), sitABCD, qacE* | *gyrA* (S83L), *gyrA* (D87Y), *parC* (S80I), *parE* (I355T) | Col8282, IncB/O/K/Z, IncFIB(AP001918), IncFII(pSFO), IncQ1, p0111 |
| 26 (ST1485; CC648) | *aadA5, aph(4)-Ia, aac(3)-IV, blaCTX-M-65, mph(A), sul1, sul2, dfrA17, floR, tet(A), sitABCD, qacE* | *gyrA* (S83L), *parC* (S80I) | IncB/O/K/Z, IncFIA, IncFIB(AP001918), IncFIC(FII), IncY |
| 96 (ST295) | *aph(4)-Ia, aac(3)-IV, aadA1, sul1, dfrA14, sitABCD, qacE* | *gyrA* (S83L), *gyrA* (D87N), *parC* (S80I) | Col8282, ColpVC, IncB/O/K/Z |
| 215 (ST351) | *aph(6)-Id, aph(3'')-Ib, blaCTX-M-55, blaTEM-214, blaTEM-209, blaTEM-206, blaTEM-141, blaTEM-1B, sul2, floR, tet(A)* | *gyrA* (S83L), *gyrA* (D87N), *parC* (S80I) | IncFII(pHN7A8), IncI1-I(Alpha) |
| 574 (ST18519*) | *aph(6)-Id, aph(3'')-Ib, blaCTX-M-55, blaTEM-214, blaTEM-209, blaTEM-206, blaTEM-141, blaTEM-1B, qnrB19, sul2, dfrA14, tet(B)* | None | Col(pHAD28), IncFII(pHN7A8) |

*New ST

**Table S3**. Genomic characterization of the three *K. pneumoniae* isolates based on whole-genome sequencing.

| **Strain ID (ST)** | **Resistance genotype** | **Mutations** | **Plasmid replicons** |
| --- | --- | --- | --- |
| 54 (ST4536) | *aph(6)-Id, aph(3'')-Ib, aadA2b, aadA16, aac(6')-Ib-cr, aac(3)-IId, aph(3')-Ia, oqxB, oqxA, qnrB52, blaSHV-11, blaSHV-67, blaCTX-M-15, blaTEM-1B, sul1, dfrA32, dfrA27, fosA, ere(A), mph(A), tet(A), tet(D), floR, ARR-3* | *acrR* (P161R, G164A, F172S, R173G, L195V, F197I, K201M), *ompK36* (N49S, L59V, G189T, F198Y, F207Y, T222L, D223G, N304E); *ompK36* (A217S); *ompK37* (I70M, I128M) | IncFIB(K), IncFIB(pNDM-Mar), IncFII(K), IncHI1B(pNDM-MAR) |
| 569 (ST323) | *aac(3)-IId, aadA1, oqxB, oqxA, qnrE1, blaSHV-193, blaSHV-178, blaSHV-80, blaSHV-36, blaCTX-M-15, dfrA32, fosA6, fosA7, ere(A), formA, qacH* | *acrR* (P161R, G164A, F172S, R173G, L195V, F197I, K201M), *ompK36* (N49S, L59V, F207W, D224E, T254S); *ompK36* (A217S, N218H); *ompK37* (I70M, I128M) | IncFIB(pNDM-Mar), IncHI1B(pNDM-MAR) |
| 636 (ST307) | *aadA1, aph(6)-Id, aph(3'')-Ib, aac(6')-Ib-cr, oqxB, oqxA, qnrB1, blaSHV-106, blaSHV-28, blaCTX-M-15, blaTEM-1B, blaOXA-1, sul2, dfrA14, fosA6, ere(A), tet(A), catB3* | *acrR* (P161R, G164A, F172S, R173G, L195V, F197I, K201M), *ompK36* (N49S, L59V, A183S, F207Y, D223G, L229V); *ompK37* (I70M, I128M, N230G, E244D) | ColpVC, IncFIB(K), IncFII(K) |

**Table S4**. Summary of antimicrobial resistance profiles among bacterial isolates recovered from broiler carcasses.

| **Species** | **Number of isolates (n)** | **MDR (%)** | **ESBL (%)** | **AmpC (%)** |
| --- | --- | --- | --- | --- |
| *Acinetobacter baumannii* | 14 | 14.3 | - | - |
| *Enterobacter* spp. | 53 | 83 | - | - |
| *Escherichia coli* | 99 | 84.8 | 56.6 | - |
| *Klebsiella pneumoniae* | 68 | 47.1 | 13.2 | - |
| *Pseudomonas aeruginosa* | 59 | 0 | - | - |
| *Salmonella* sp. | 48 | 52.1 | 52.1 | 52.1* |
| *Staphylococcus aureus* | 10 | 50 | - | - |

*AmpC production was assessed only for *Salmonella* isolates.

**Table S5**. Genome assembly quality metrics

| **Strain** | **Bacteria species** | **Total assembly length (bp)** | **N50 (bp)** | **Number of contigs** | **GC content (%)** | **Estimated sequencing depth (×)** |
| --- | --- | --- | --- | --- | --- | --- |
| 44 | *Salmonella* Minnesota | 4,818,284 | 224,921 | 71 | 52.15 | 106 |
| 59 | *Salmonella* Minnesota | 4,857,296 | 219,338 | 72 | 52.14 | 87 |
| 165 | *Salmonella* Minnesota | 4,851,651 | 219,321 | 73 | 52.14 | 96 |
| 618 | *Salmonella* Minnesota | 4,855,797 | 219,321 | 66 | 52.14 | 105 |
| 9 | *Escherichia coli* | 5,664,924 | 134,779 | 231 | 50.48 | 92 |
| 26 | *Escherichia coli* | 5,291,310 | 363,360 | 86 | 50.47 | 103 |
| 96 | *Escherichia coli* | 5,596,395 | 97,263 | 263 | 50.47 | 100 |
| 215 | *Escherichia coli* | 4,958,969 | 164,020 | 93 | 50.58 | 113 |
| 574 | *Escherichia coli* | 4,614,403 | 106,759 | 124 | 50.79 | 102 |
| 54 | *Klebsiella pneumoniae* | 5,767,699 | 214,308 | 100 | 56.84 | 83 |
| 569 | *Klebsiella pneumoniae* | 5,482,100 | 273,403 | 73 | 57.02 | 83 |
| 636 | *Klebsiella pneumoniae* | 5,596,417 | 254,315 | 94 | 57.17 | 97 |
