# Supplementary material for: Antimicrobial resistance and genomic characterization of Salmonella Minnesota, ESBL-producing Escherichia coli, and ESKAPE pathogens isolated from Brazilian broiler chicken carcasses
Source: Front Microbiol. 2026 Sep 15;17:1917698. doi: 10.3389/fmicb.2026.1917698 (PMC13619903; doi:10.3389/fmicb.2026.1917698)
Supplement: Supplementary file 2 [file Table_3.DOCX]

Supplementary Material 3

**Table S3**.**1** Plasmid replicons and associated resistance genes in *Salmonella* Minnesota sequenced isolates.

| **Strain ID** | **Plasmid replicons** | **Antimicrobial resistant determinant** |
| --- | --- | --- |
| 44 | Col440I | *qnrB19* |
|  | IncA/C2 | *aadA1*, *aph(3')-Ia*, *sul2*, *tet(A)* |
| 59 | Col440I | *qnrB19* |
|  | IncA/C2 | *aadA1*, *aph(3')-Ia*, *sul2*, *tet(A)* |
| 165 | Col440I | *qnrB19* |
|  | IncA/C2 | *aadA1*, *aph(3')-Ia*, *sul2*, *tet(A)* |
| 618 | Col440I | *qnrB19* |
|  | IncA/C2 | *aadA1*, *aph(3')-Ia*, *sul2*, *tet(A)* |

**Table S3.2**. Plasmid replicons and associated resistance genes in *Escherichia coli* sequenced isolates.

| **Strain ID** | **Plasmid replicons** | **Antimicrobial resistant determinant** |
| --- | --- | --- |
| 9 | IncFIB(AP001918) | sitABCD |
|  | IncB/O/K/Z | aac(3)-IV, blaCMY-2, sul2 |
|  | IncFII(pSFO) | ND |
|  | p0111 | ND |
|  | Col8282 | ND |
|  | IncQ1 | aph(3')-Ia, fosL1 |
| 26 | IncFIA, IncFIB(AP001918), IncFIC(FII) | tet(A), sitABCD |
|  | IncB/O/K/Z | aph(4)-Ia, aac(3)-IV, blaCTX-M-65, sul2 |
|  | IncY | ND |
|  | ND | aadA5, sul1, dfrA17, mph(A), floR, qacE |
| 96 | IncB/O/K/Z | aac(3)-IV, aph(4)-Ia |
|  | ND | sul1, qacE |
|  | ColpVC | ND |
| 215 | IncFII(Phn7A8) | aph(3’’)-Ib, aph(6)-Id, blaCTX-M-55, blaTEM-214, blaTEM-209, blaTEM-206, blaTEM-141, blaTEM-1B, sul2, fosA3, tet(A), floR |
|  | IncI1-I(Alpha) | ND |
| 574 | IncFII(Phn7A8) | blaCTX-M-55, blaTEM-214, blaTEM-209, blaTEM-206, blaTEM-141, blaTEM-1B |
|  | Col(Phad28) | qnrB19 |
|  | ND | aph(6)-Id, aph(3’’)-Ib, sul2, dfrA14 |

*ND = Not detected

**Table 3**. Plasmid replicons and associated resistance genes in *Klebsiella pneumoniae* sequenced isolates.

| **Strain ID** | **Plasmid replicons** | **Antimicrobial resistant determinant** |
| --- | --- | --- |
| 54 | IncFIB(pNDM-Mar) | aac(3)-IId, aadA2b, aph(3')-Ia, blaCTX-M-15, blaTEM-1B, sul1, dfrA32, ere(A), mph(A), tet(D) |
|  | IncFIB(K), IncFII(K) | aph(6)-Id, aph(3'')-Ib, aph(3'')-Ib, aph(3'')-Ib, aph(3'')-Ib, tet(A) |
|  | ND | qnrB52, floR |
| 569 | IncFIB(pNDM-Mar) | aac(3)-IId, aadA1, qnrE1, blaCTX-M-15, dfrA32, ere(A), formA, qacH |
| 636 | IncFIB(K), IncFII(K) | aac(6')-Ib-cr, aph(6)-Id, aph(3'')-Ib, qnrB1, blaCTX-M-15, blaTEM-1B, blaOXA-1, sul2, dfrA14, tet(A), catB3 |
|  | ColpVC | ND |
|  | ND | aadA1 |

*ND = Not detected
