# Supplementary material for: Antimicrobial resistance and genomic characterization of Salmonella Minnesota, ESBL-producing Escherichia coli, and ESKAPE pathogens isolated from Brazilian broiler chicken carcasses
Source: Front Microbiol. 2026 Sep 15;17:1917698. doi: 10.3389/fmicb.2026.1917698 (PMC13619903; doi:10.3389/fmicb.2026.1917698)
Supplement: Supplementary file 3 [file Table_5.DOCX]

Supplementary Material 5

**Table S5** Sequencing and assembly details of *Salmonella* Minnesota (n=4), *Escherichia coli* (n=5) and *Klebsiella pneumoniae* (n=3) strains recovered from broiler chicken carcasses.

| **Strain** | **Bacteria species** | **Total assembly length (bp)** | **N50 (bp)** | **Number of contigs** | **GC content (%)** | **Estimated sequencing depth (×)** |
| --- | --- | --- | --- | --- | --- | --- |
| 44 | *Salmonella* Minnesota | 4,818,284 | 224,921 | 71 | 52.15 | 106 |
| 59 | *Salmonella* Minnesota | 4,857,296 | 219,338 | 72 | 52.14 | 87 |
| 165 | *Salmonella* Minnesota | 4,851,651 | 219,321 | 73 | 52.14 | 96 |
| 618 | *Salmonella* Minnesota | 4,855,797 | 219,321 | 66 | 52.14 | 105 |
| 9 | *Escherichia coli* | 5,664,924 | 134,779 | 231 | 50.48 | 92 |
| 26 | *Escherichia coli* | 5,291,310 | 363,360 | 86 | 50.47 | 103 |
| 96 | *Escherichia coli* | 5,596,395 | 97,263 | 263 | 50.47 | 100 |
| 215 | *Escherichia coli* | 4,958,969 | 164,020 | 93 | 50.58 | 113 |
| 574 | *Escherichia coli* | 4,614,403 | 106,759 | 124 | 50.79 | 102 |
| 54 | *Klebsiella pneumoniae* | 5,767,699 | 214,308 | 100 | 56.84 | 83 |
| 569 | *Klebsiella pneumoniae* | 5,482,100 | 273,403 | 73 | 57.02 | 83 |
| 636 | *Klebsiella pneumoniae* | 5,596,417 | 254,315 | 94 | 57.17 | 97 |
